# Depression in adolescence and the understanding of health—A phenomenographic study

**DOI:** 10.1371/journal.pone.0318061

**Published:** 2025-01-27

**Authors:** Klara Danielsson, Mikael Ahlborg, Rebecca Mortazavi, Håkan Jarbin, Ingrid Larsson

**Affiliations:** 1 Department of Health and Care, School of Health and Welfare, Halmstad University, Halmstad, Sweden; 2 Department of Paediatric Care, Uppsala University Hospital, Section of Obesity in Children and Adolescents, Region Uppsala, Uppsala, Sweden; 3 Child and Adolescent Psychiatric Clinic, Region Halland, Halmstad, Sweden; 4 Faculty of Medicine, Department of Clinical Sciences, Child and Adolescent Psychiatry, Lund University, Lund, Sweden; 5 Spenshult Research and Development Centre, Halmstad, Sweden; 6 Department of Clinical Sciences, Section of Rheumatology, Lund University, Lund, Sweden; Harvard University HSPH: Harvard University T H Chan School of Public Health, UNITED STATES OF AMERICA

## Abstract

Health is multifaceted, with divergent interpretations in diverse cultures and groups of individuals. The ways individuals understand health might aid in developing future interventions. There is scant knowledge on how adolescents with depression conceptualise health. A descriptive qualitative design with a phenomenographic approach was used to describe the different ways adolescents with depression conceptualise health. Interviews were performed with adolescents 13–17 years old (n = 33) who participated in a randomised controlled trial evaluating the effects of aerobic group exercise versus leisure group activities for adolescents with depression. The results were interpreted into four metaphors to embody the understanding of health as described by adolescents with depression: 1) establishing sound routines by managing everyday life, 2) connecting to others by having access to social resources, 3) managing depression symptoms by having control over the mental illness, and 4) attaining inner drive by experiencing joy in everyday life. The various conceptions of health among adolescents with depression provide valuable insights for enhancing evidence-based treatments with person-centred care. Key aspects include establishing routines, fostering connections, finding strategies for symptom control, and incorporating joy through exercise. Given that adolescents highlighted these aspects as essential to health, future research could explore individualised health promotion, particularly focusing on routine-building, social connections, or finding an inner drive as an add-on to evidence-based treatments for adolescent depression.

## Introduction

The rise in adolescent depression constitutes a global public health concern [1–4]. While self-reported depressive symptoms have undeniably increased among adolescents [3,4], the incidence of depression varies depending on sampling, definition and diagnostic instrument, and time frame [5]. When looking at past-year prevalence of Major Depressive Disorder and Major Depressive Episode (DSM-5), incidence ranges between 8.8% [6] and 13.8% [7] in European adolescents and 7.5% [8] to 12.9% [9] in US adolescents. These statistics translate into about 1 in 10 adolescents going through a major depressive episode each year. The prevalence increases with age, and females are increasingly overrepresented [2,9,10]. Recent studies reveal additional increases in depressive symptoms among adolescents during the COVID-19 pandemic possibly due to the restrictions that followed [7,11–13].

A depressive episode is characterised by persistent sadness, irritability and/or anhedonia lasting for a duration of at least two weeks concurrent with somatic and cognitive symptoms [14]. The symptoms can, therefore, be cognitive, behavioural, or somatic and must persist over time. Depression can be categorised as mild to severe, depending on the intensity of the symptoms and the extent to which it affects everyday functions. Children and adolescents diagnosed with depression can face a long course of illness that requires psychiatric care and drug treatment [15]. Depression is associated with an increased risk of suicide, substance misuse, obesity [16], and self-harm [17]. Additionally, adolescents with depression are significantly more likely to suffer from depression in adulthood compared to their peers [4,18].

Adolescence is defined as the period between 10 and 19 years, during which rapid changes occur in physical, cognitive, and psychosocial abilities [19]. This affects an individual’s emotional life, thinking, decision-making, and social interaction. Parallel to this development, the foundations of health are being formed [19]. Recent studies indicate that adolescents from the general population perceive health in terms of physical appearance, commitment, goals, resources, space, use of free time, social belonging, healthy diet, and physical activity [20,21]. Additionally, adolescents emphasise the importance of having trusting and supportive relationships in maintaining their mental well-being [22–24].

The concept of health is multifaceted and can have varying meanings between different cultures and individuals [25]. Meanwhile, there is a link between subjective health perception, health behaviours and depressive symptom trajectory from adolescence into young adulthood [26]. This link highlights the importance of addressing both risk and protective factors in mental health research and practice, while recognizing the complexities of adolescent development. Protective factors can be described as conditions or attributes that mitigate negative effects of stressful life events or enhance an individual’s ability to avoid risk behaviours [20]. The presence of protective factors can, in turn, decrease the incidence and prevalence of known risk factors [21]. While there is a multitude of known risk factors, e.g. heredity, substance use, dieting, negative coping strategies etc. [27], there are comparatively fewer protective factors that adolescents can actively modify. Key protective factors include self-confidence, social competence, support and family cohesion [28]. These factors are crucial for mental well-being but typically require sustained intervention and professional support to bring sustainable change.

In comparison, health behaviour change may offer a more immediate and tangible area for intervention. Still, prior to implementing therapeutical interventions, it is essential to explore health from the perspective of adolescents with depression for multiple reasons. First, studies have examined adolescents’ perspectives on health [20,21]. However, there remains a dearth of research specifically focusing on adolescents with depression. Second, engaging adolescents with depression in the development of interventions tailored to their needs is crucial [24] for bridging the research gap and creating feasible implications for practice [29]. Lastly, it is essential to provide a platform for adolescents with depression to empower them to be active agents in their mental health journey and to recognize their perspective in mental health research.

### Aim

The study’s aim was to describe different ways of understanding health among adolescents with mild to moderate depression.

## Method

### Design

A descriptive qualitative design with a phenomenographic approach was chosen to describe the different ways adolescents with depression understand the phenomenon of health. A phenomenographic approach identifies variations in conceptions of a specific phenomenon within a group, revealing that multiple ways of understanding can coexist among individuals [30]. In order to grasp adolescents’ understanding of health, it is crucial to recognize the distinctions in their perceptions. Within the phenomenographic approach, both the what-aspect and the how-aspect are included. The what-aspect informs us about the specific focus of the adolescents’ understanding, while the how-aspect describes how meaning is constructed regarding health. These different descriptions of health form categories that encompass various aspects of how adolescents with mild to moderate depression understand health. The outcome space in phenomenography captures these categories, illustrating the range of ways in which participants conceptualize health and highlighting the relationships between different understandings [31]. Ultimately, this approach allows for a deeper insight into the nuanced experiences of these adolescents, facilitating a better understanding of their health-related perspectives and informing interventions aimed at promoting their well-being. The study followed the consolidated criteria for reporting qualitative research (COREQ 32)—item checklist [32].

### Setting

This qualitative study was part of a randomised controlled trial (RCT) with trial registration number NCT05076214, and a preceding pilot study conducted in Sweden [33]. The RCT aimed to compare the effects of group aerobic exercise to group leisure activities in adolescents diagnosed with depression. Adolescents were included from December 1^st^, 2020, through February 26^th^, 2021, for the pilot RCT and from January 31^st^, 2022, through February 25^th^, 2022, for the main RCT. The adolescents were randomised to one of the two groups and met for sessions 3 times a week for 12 weeks. Adolescents were recruited from four Child and Adolescent Mental Health Services in Sweden. As a part of the project, qualitative interviews were conducted with the participants after the intervention period [33].

The inclusion criteria for participation in both the pilot study or multicentre RCT were adolescents: 1) aged 13–17 years with mild-to-moderate depression according to the DSM-5, 2) who had attended at least three clinical visits and received some brief psychosocial intervention (BPI) for depression, and 3) had not shown a clear response to previous treatment (BPI, psychotherapy or medications), that is at least 50% reduction of depressive symptoms.

The exclusion criteria for participation were: 1) eating disorder; 2) at high risk of suicide, which would necessitate adjustment of medication or psychotherapeutic interventions; 3) intellectual disability; 4) ongoing physical exercise at least 75 minutes per week of intense exercise or 150 minutes per week of moderate-intensity; 5) recently adjusted medication; 6) ongoing psychotherapeutic treatment; and 7) in need of an interpreter [33].

### Sample

The sample consisted of 33 adolescents diagnosed with mild to moderate depression included in the RCT or pilot study who had participated in a 12-week group intervention with aerobic exercise or leisure activities. Nine adolescents completed the pilot study, and all participated in qualitative interviews. The RCT included 35 participants at the time of this qualitative study. All 35 were invited by phone to participate in qualitative interviews, and 24 chose to participate. The adolescents who declined to participate did so citing competing commitments, such as academic obligations. The sample had variation regarding age, gender, and degree of depression (Table 1).

**Table 1:** Overview of the demographics of the adolescents.

Variables	Adolescents (n = 33)
**Age** Years (median (range))	16 (13–17)
**Sex** (n) Female Male	24 9
**CDRS-R baseline**^**1**^ (median score (range))	55 (32–75)
**QIDS-A17-C baseline**^**2**^ (median score (range))	15 (6–23)
**CGAS baseline**^**3**^ (median score (range))	45 (31–70)
**Duration of depression** (median months (range))	26 (2–103)
**Comorbidity with ADHD**^**4**^ (n (percent))	19 (57.6)
**Comorbidity with anxiety disorder** (n (percent))	19 (57.6)
**12-week group intervention** (n) Exercise Leisure activity	16 17

^1^ Children’s Depression Rating Scale-Revised.

^2^ Quick Inventory of Depressive Symptomatology—Adolescent version-17 Clinician rated.

^3^ Children Global Assessment Scale.

^4^ Attention Deficit Hyperactivity Disorder.

### Data collection

Data were collected through semi-structured interviews. The interviews were held in Swedish by an independent, experienced researcher in qualitative research (I.L.) or by trained and supervised research fellows with experience in child and adolescent psychiatry (R.A. & E.H.). The interviews took place between the summer of 2021 and the fall of 2022, via telephone or the digital tool Zoom. This made it possible for the adolescents to decide where the interviews were to be held or if they wished for a parent to participate. Most of the interviews were conducted via telephone, with the adolescent participating from their home. Examples of questions were “What is health to you?”,” How do you perceive your health?” and “What do you do to care for and promote your health?”. The interview guide was developed for the pilot study and RCT on exercise for depression and can be found as a supplementary file in the study protocol [33]. We conducted two pilot interviews that were included in this study since no changes of the interview guide were necessary. The interviews were audio recorded and lasted between 30 and 72 minutes, with a total interview length of 29 hours and 3 minutes. All interviews were transcribed verbatim.

### Data analysis

**Fig 1:**
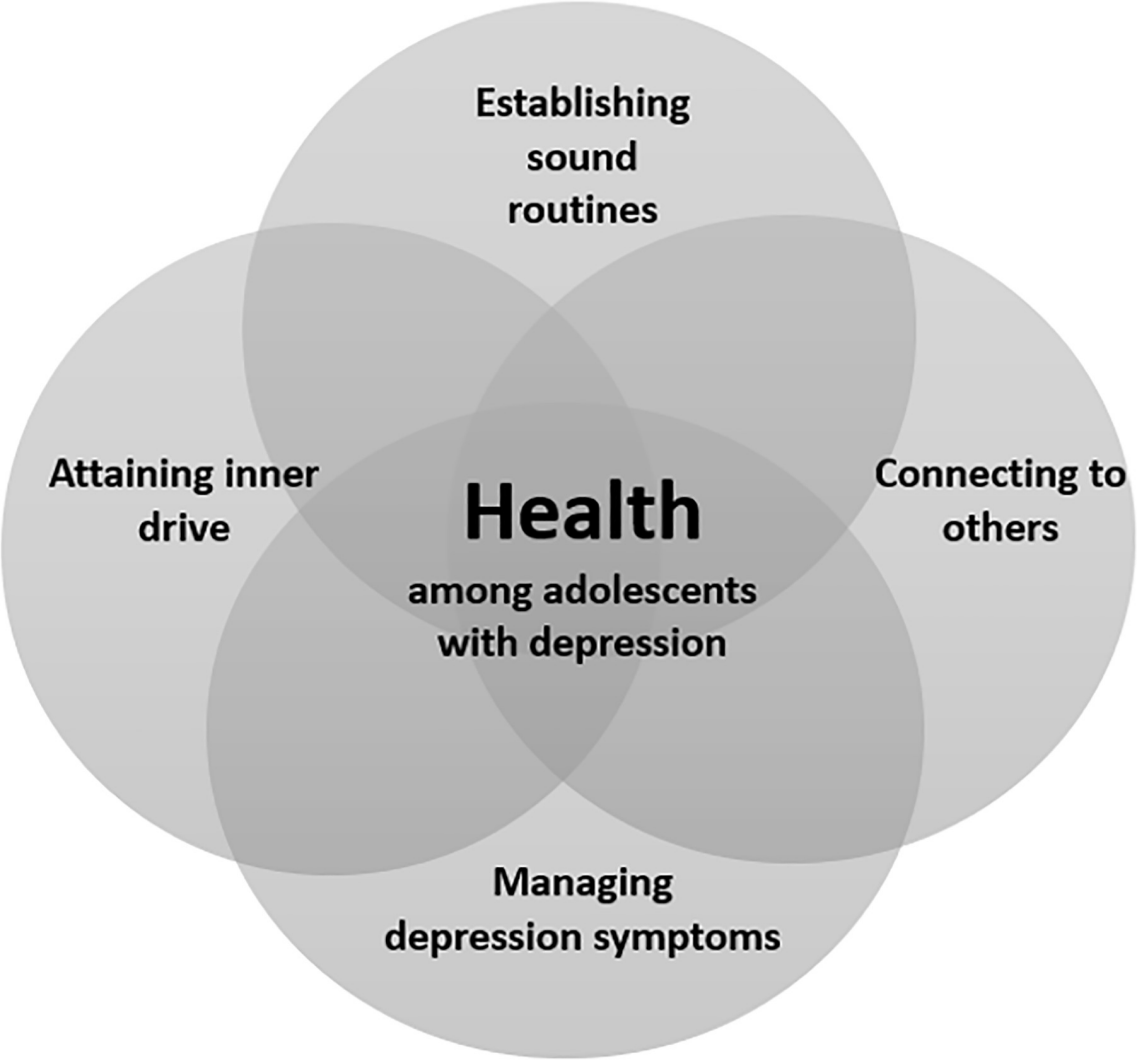
Overview of the outcome space, the collective understanding of health among adolescents with depression. The figure visualizes the relationship between the four metaphors.

In phenomenography, developed by Ference Marton et al., data analysis involves a two-stage process. The first stage focuses on identifying and describing the overall meanings of participants’ conceptions by segmenting transcripts into meaning units, with a new unit formed each time a distinct meaning is expressed. In the second stage, the analysis identifies the structural aspects of each conception. Each meaning unit is examined to discern which elements of the phenomenon participants emphasize, capturing both explicit and implied variations. This process results in the construction of categories of description that represent the range of conceptions of the phenomenon and their internal relationships, i.e., the outcome space [30,34]. In this study, data analysis followed Marton et al.’s two-stage process and was performed in seven steps, following the phenomenological analysis introduced by Larsson and Holmström (2007): 1) transcripts were read thoroughly to become familiar with the empirical material; 2) the entire text was reread, and conceptions on health given by each adolescent were identified and marked; 3) a preliminary description of each adolescent’s dominant way of understanding health was formulated by examining their main areas of focus and assessing how central this understanding of health was to their overall perspective, as reflected by the participants’ tendency to return to this description multiple times throughout each interview; 4) descriptions of health were then grouped based on similarities and differences in meaning, which resulted in four descriptive categories, and these categories were then compared to establish that each had a unique character and the same level of description; 5) a search for non-dominant ways of understanding the phenomenon of health was undertaken to ensure that no aspect was overlooked; 6) a structure of descriptive categories; i.e., the outcome space of the study, was created to describe the relationship between categories (Fig 1); 7) finally, a metaphor was assigned to each descriptive category emanating from joint interpretation and discussions after categories were decided upon.

The first author (K.D.) conducted the first analysis and coded the data with M.A. and I.L. as co-assessors. The analysis was discussed with the entire research group until a consensus was reached. The authors have extensive experience in research about adolescents’ health and qualitative methodology. The quotations included in the manuscript were translated from Swedish to English by a certified translator.

### Ethics

The study has been approved by the Swedish ethical review authority (RCT: No. 2021-05307-01; pilot study: No. 2020–03364). For the study to be carried out according to ethical principles, the ethical guidelines set out in the Declaration of Helsinki have been followed, including information, consent, confidentiality, and benefit [35].

Both adolescents and parents received thorough written and verbal information about the study. For both the pilot study and multicentre RCT, according to the ethical review authority, consents in writing were collected from participants 15 years and above and from guardians to participants 13–14 years. Oral consent was obtained from all participants and from parents.

Adolescents were informed about voluntary participation and their ability to withdraw from the pilot study and multicentre RCT at any time without offering any explanation.

## Results

Adolescents with depression described different ways of understanding health. Interpreting the descriptive content led to forming metaphors to represent each of the four initial categories (Table 2). The category ‘ability to manage everyday life’ was given the metaphor *establishing sound routines*. ‘Access to social resources’ was assigned the metaphor *connecting to others*. The category ‘control over mental illness’ was given the metaphor *managing depression symptoms*. Lastly, the category ‘experience joy in everyday life’ was assigned the metaphor *attaining inner drive*.

**Table 2:** Overview of the descriptive categories with associated metaphors describing how adolescents with depression understand health.

Categories	*Metaphors*
**Ability to manage everyday life**	*Establishing sound routines*
**Access to social resources**	*Connecting to others*
**Control over mental illness**	*Managing depression symptoms*
**Experience joy in everyday life**	*Attaining inner drive*

### Establishing sound routines

Health was described as establishing sound routines, meaning having the ability to manage everyday life and having the capacity to be able to get through the day without unobtainable obstacles. This included maintaining healthy lifestyle habits, following routines, feeling inner and outer balance, and engaging in distracting activities. Having these routines and strategies enabled the adolescents to manage their everyday life, and thereby maintaining their health.

Adolescents described sound routines as having a good balance between lifestyle habits such as diet, physical activity, and sleep. The adolescents perceived that physical activity affected physical and mental health, but there was a variation where some adolescents perceived that physical activity only affected physical health. Maintaining healthy lifestyle habits could also be seen as a way of maintaining a functioning circadian rhythm, which was considered essential in coping with everyday life. One of the adolescents articulated the following:

*“Yes. It’s like something that’s constant. It’s just like having a good relationship with food and it is sort of like a trinity with sleep, food, and exercise, I think. It is important to keep them on track (…)”*

      (Adolescent no. 17)

The adolescents perceived that establishing routines was part of managing everyday life and associated with health. Sound routines served as a source of motivation, prompting the adolescents to get up in the morning, leave home, and attend school. In addition, the routines could be considered more important than the actual activity or task itself. Routines empowered adolescents to navigate their day with a clear understanding of the planned activities and the associated expectations. Organising tasks within routines provided a sense of structure, enabling adolescents to feel in control of their everyday lives. This organisational approach was perceived as an integral aspect of maintaining health and was described as following by one of the adolescents:

*“I think I have gone to school more when I have been to those groups because I get up in the morning, I have a routine, I can socialise more, and I can think better. Much more clearly. That’s probably good.”*

      (Adolescent no. 18)

Maintaining an inner and outer balance between physical and mental factors as well as external circumstances, was a way of managing everyday life. The adolescents described how a connection between body and mind was a part of experiencing health. Furthermore, external factors, such as financial stability, contributed to a more stable and balanced environment. Achieving a sense of inner and outer balance involved engaging in activities that the adolescents typically enjoyed, as well as effectively managing daily tasks. It was acknowledged that feeling down at times was a natural part of life; however, it should not escalate to the point where it hindered the management of daily responsibilities such as schoolwork and leisure activities. The adolescents wished to have a stable emotional state where peaks and valleys were balanced and moderate. The following was expressed:

*“So, you feel good both in your head and in your body. So, you feel good. Then you might have ups and downs, but that’s also what makes you feel good. That you should both have a little up and down, but it should still be stable in both. You shouldn’t feel too much of the bad and you shouldn’t feel too much of the good either, everything should be stable.”*

      (Adolescent no. 29)

Furthermore, engaging in distracting activities was essential to managing everyday life and, therefore, a crucial aspect of health. Activities such as work, leisure activities, or hanging out with friends gave adolescents a break from reality or thoughts and feelings that could arise during the day. Activities served a dual purpose; some were employed to fill time, while others were deliberately chosen for mindful engagement. Some adolescents adapted this practice more consistently, recognising it as an effective strategy for establishing sound routines to maintain health. The activities provided a temporary respite, allowing adolescents to escape difficult, challenging emotions. One of the adolescents described this in the following manner:

*“Maybe play games with my friends. I think it’s probably the most fun. You can get involved with your friends and just have fun. And don’t think about anything else, just your friends and the game.”*

      (Adolescent no. 9)

### Connecting to others

Connecting to others was described as having access to social resources, which was considered an essential aspect of health. Thus, having social support and experiencing connectedness could prevent feelings of isolation. From this perspective, health was described as experiencing social support, social competence, and connecting to others.

Having a family member or friend who was aware of and understood the adolescents’ situation regarding their depression played a vital role in their health. Additionally, pets were acknowledged for providing crucial support, characterised by their non-judgmental nature and capacity for unconditional companionship. On the other hand, a lack of social support was linked to feelings of isolation, loneliness, and deteriorating health. Moreover, social support was described as experiencing love and care along with the ability to openly share thoughts and emotions with friends and family. One of the adolescents described the importance of friends and family in the following way:

*“So, if you have no friends, a feeling that you have a bad relationship with your family, no one to talk to, in my case, you become very isolated and very withdrawn, then you can suffer from depression or a bad feeling or worsened health because of it.”*

      (Adolescent no. 32)

Social competence was described as the capacity to feel comfortable and independent in diverse social settings, enabling open communication and interaction as part of a social resource. For some adolescents, it was important to have the opportunity to express opinions regarding, for example, politics, without being afraid of the response. Other adolescents expressed social anxiety and described a feeling of panic when they were forced to socialise. According to adolescents, possessing social competence could mitigate this anxiety and was consequently seen as an integral aspect of health. Having developed social competence empowered them to engage with others, avoid isolation, and cultivate new relationships, thus enhancing connectedness with others and their overall health. This was described as:

*“For me, it has meant that there is another chance for me to feel better. Find new strategies and such, what can help me and such. It has meant a lot because I have a bit of a problem with social things sometimes and then it has still given me some good knowledge or something. About getting to know new people, being able to talk and be involved, and so on.”*

      (Adolescent no. 7)

Feelings of social connectedness were evident in environments where adolescents could interact and be integrated into a social community. The adolescents described how social connectedness could manifest in different settings like school, work, or leisure activities, such as online gaming, or watching movies where the adolescent felt connected to one or several characters. Nevertheless, social settings could adversely affect adolescents when they perceived a sense of disconnectedness. This became pronounced when they felt insecure or were involved in a disagreement with friends, potentially resulting in feelings of exclusion and fear of not belonging. Connectedness, perceived as a valuable social resource in daily life, was an essential aspect of health—especially when there were opportunities to connect and share common interests with like-minded individuals. This was described in the following way by one of the adolescents:

*“I didn’t feel any difference in how my daily health was. So, I just felt okay, I’ll go there, I’ll go home and then it’s done. But after that. Like it’s starting to be a little fun to go there. It was fun to be able to talk to someone who has the same thoughts and hobbies. I was with another girl, so we had a lot in common. We talked and it was fun.”*

      (Adolescent no. 8)

### Managing depression symptoms

Managing the symptoms of depression meant that adolescents searched for treatments and strategies to avoid experiencing the symptoms of depression. The adolescents described health as feeling in control of their depression, such as feeling relief from symptoms, having effective strategies or medication, and experiencing feelings of well-being. This ongoing and often frustrating process revealed how they compared themselves to others.

Feeling relief from symptoms was described as not suffering from the symptoms associated with depression. Adolescents perceived that maintaining good health was difficult while dealing with depressive symptoms. Illness was perceived as a result of suffering from depression. This perspective conveyed that the symptoms of depression were at odds with a state of well-being, emphasising the necessity of managing these symptoms to effectively attain a sense of health. This was described by one of the adolescents in the following way:

*“Well, a lot would have to be different, I should stop having so much anxiety, stop having so much depression, stop having social anxiety. There is a lot that I need to stop having.”*

      (Adolescent no. 16)

The adolescents perceived that effective strategies were required to manage their depressive symptoms. However, it was not always clear to them whether pharmacological or non-pharmacological strategies were more appropriate. Some perceived that many treatment methods probably worked for others but not for them, and some conveyed that they had not received the help they needed from professionals. Some adolescents explained that they had tried everything from medication to physical activity to writing in a diary and that there were now no options left to get rid of the depression and thus perceive health. A clear illustration of this can be seen in the quotation below:

*“Medication, that kind of talk, CBT, or whatever it’s called, exercise, eating well, going out for a walk every day, (…) having time for me to be with my family and friends and stuff like that. So, I force myself to do everything. I sit and google almost every day what I can do to feel good. I’ve tried writing in a diary, I’ve tried, I have like six minutes a day when you should only write down positive things that have happened during the day. I’ve tried everything and I don’t know anymore.”*

      (Adolescent no. 4)

Furthermore, experiencing general health was described as physical and especially mental well-being. Well-being was described as a subjective feeling where adolescents were satisfied with how they felt in body and mind. It could also be linked to not having depression. According to adolescents, well-being was an essential aspect of health, as described in the following quotation:

*“(…) most of what I think about is well-being, that is the most important thing when it comes to health (…)”*

      (Adolescent no. 1)

### Attaining inner drive

Attaining an inner drive was described as experiencing joy in everyday life and characterised by a predominant feeling of zest for life, containing a profound sense of meaning and self-worth. The experience of joy was also intertwined with having a sense of purpose, inner drive, and self-compassion. Having a sense of purpose was described as a feeling of significance in life, whether this meant having a goal, a dream, something to look forward to, or finding meaning in leisure activities, work, or through connection with friends and family. This sense of purpose motivated adolescents to get up in the morning and carry them through the day. The adolescents emphasised the importance of daily accomplishment to feel a sense of purpose and was described by one adolescent:

*“It was like something to do during the day, so it affected my mood quite a lot (…) Yes, but I felt happier as if I was there and achieved something during the day, like (…) Hmm, because before it was like when I felt like my worst, I’ve just been lying in bed all day. And then when you go to bed, you get this anxiety that you haven’t done anything all day, so it’s nice to sort of get something done.”*

      (Adolescent no. 6)

Feeling an inner drive was described as an inner desire, such as motivation, energy, or joy. Motivation and energy were something adolescents lacked during their depressive episodes. A lack of inner drive resulted in not having the energy to do things they usually liked to do and difficulties in attending school. Thus, an inner drive was described as an essential aspect of health. Adolescents described the importance of experiencing desire for various moments or tasks, as it invigorated them and provided the necessary energy for these activities. One adolescent described this desire in the following way:

*” (…) While I was less depressed and sad, it was just that I was able to do things more. I had a little more interest in doing things (…) I was able to participate more in school or hang out with people, do activities and things like that (…) Listen and work and stuff like that and just be there because I wouldn’t have been able to before.”*

      (Adolescent no. 27)

The adolescents described that self-compassion entails perceiving themselves in a positive and affirming light without undermining themselves, their thoughts, or their emotions. An aspect of self-compassion included allowing themselves to live in a way that encompasses self-care, feeling comfortable in their own skin, positive self-talk, and avoiding unhealthy comparisons with others. Moreover, adolescents perceived prioritising their well-being to be essential, even though they recognised the challenges associated with prioritising themselves over others. An adolescent’s perceived importance to prioritise well-being was captured in the following quotation:

*“(…) You must take care of yourself. And think about how you feel. And what I should do to feel better.”*

      (Adolescent no. 11)

### The outcome space

The outcome space consists of the relationships between the four metaphors describing the understanding of health among adolescents with depression (Fig 1). This has been developed and interpreted based on the collective varying understanding and does not represent individual differences. The interrelated categories form parts of a whole that are not hierarchically ordered. They rely on each other in different ways and at varying levels, capturing the diversity in how health can be understood by adolescents facing depression.

For some adolescents, health may be represented by two or three of these metaphors, while for others, it may involve all four. Together, the four metaphors, establishing sound routines, connecting to others, managing depression symptoms, and attaining inner drive, comprise the variations in how adolescents describe health when struggling with depression.

Establishing sound routines depends on the other three metaphors and can, therefore, be facilitated through connecting to others, managing depression symptoms, and attaining inner drive. Similarly, connecting to others can be facilitated by establishing sound routines, managing depression symptoms, and attaining inner drive, making the categories co-dependent. Each metaphor can, in turn, support the others, forming the outcome space that collectively represents the different ways adolescents understand health when living with depression. This mutual reinforcement is illustrated by the intersecting circles in Fig 1, symbolizing the interconnected, collective understanding of health within this group.

## Discussion

In this qualitative study, adolescents with depression perceived health as consisting of establishing sound routines, connecting to others, managing symptoms, and attaining inner drive. This meant managing everyday tasks, being able to access social resources, feeling in control of their mental illness, and feeling joy in everyday life.

An essential aspect of health was establishing sound routines, including maintaining healthy lifestyle habits, which was a crucial aspect of managing everyday life. Routines were perceived as “beneficial” or “health-promoting” as they motivated individuals to rise from bed and acted as a diversion from self-criticism. Some adolescents suggested that diet, sleep, and everyday physical activity were synonymous with health, and thus could act as an essential component of their treatment and recovery from depression. However, not all adolescents acknowledged the link between physical activity and mental health. In previous studies, adolescents from the general population have described that a healthy individual should be in good physical shape [20,21]. Establishing healthy lifestyle habits during adolescence is particularly important, since they are likely to follow individuals into adulthood [36], and physical fitness is an essential indicator of a healthy lifestyle [37]. For adolescents in general, the pressure from parents, other adults, and society to take responsibility for one’s decisions increases with age, affecting their health depending on how this pressure is managed [38,39], and academic achievement is one such responsibility that constitutes a prominent stressor [38]. Depression further aggravates the burden of these stressors [16], through, for example, declining academic motivation [40], which accents the necessity of behaviour activation and the establishment of routines when designing interventions for adolescents [4]. Nonetheless, schools can also help increase academic motivation through high-quality education, which is related to increased well-being and a healthy transition into adulthood [38]. In our study, the adolescents seemed to equate routines with coping strategies that helped them reduce depressive symptoms through both activation and distraction.

Feeling connected to others was also described as an essential aspect of health, and this aligns with previous studies [20,23,24]. Adolescents with depression described connection to others as having access to social resources and energy to engage in social interactions. Moreover, insufficient social engagement can elevate the risk of various psychological conditions, including depression [36]. Social relationships significantly influence the habits and health-promoting or risk behaviours that develop during adolescence [36,38]. Adolescents are sensitive to managing social relationships and peer pressure, which could serve an evolutionary function to fit into an evolving social context. This relational sensitivity may explain the increased need for friendships during adolescence [36] and why social belonging and connectedness were described as essential to health. Moreover, depression in adolescence has been linked to both social isolation [41] and social exclusion [42] which highlights the role of social support for health. The studied sample was probably at a heightened risk of social isolation due to long duration of depression along with significant comorbidity with ADHD and anxiety disorders. Social withdrawal can be mitigated through well-designed interventions aimed at increasing mental health literacy and reducing stigmatisation in the general population [43]. Social engagement is, for example, frequently used in behavioural activation therapy, a part of cognitive behavioural therapy that has shown promising results for adolescents [44]. A systematic review of behavioural activation for young people with depression shows that social interventions were experienced as helpful in several qualitative studies [45], indicating that sociability may play an important role for adolescents with depression. Connecting to others, as described by the adolescents in this study, also included access to social support in their closest networks. Together, this amplifies the necessity of ensuring that people close to the adolescent are made aware of the diagnosis in general, as well as the importance of social support, so that they can encourage healthy behaviours and nurture a trusting relationship.

A third essential aspect of health among adolescents with depression was managing the symptoms of depression in order to experience relief and, accordingly, being able to achieve health. While some emphasised finding a treatment that worked for them as imperative, others experienced a sense of hopelessness when previous attempts to gain control over their symptoms had failed. Previous research highlights the role of clinicians in encouraging patients’ efforts to manage their mental illness [46]. When clinicians acknowledge the hard work and endurance of the patient in combination with a positive attitude, patients are less likely to experience hopelessness and instead feel reassured that they are on the right path toward recovery or at least toward healthier behaviours. Although this may seem obvious, depression is heterogenous and involves biological, intrapersonal, and social difficulties [47]. The various and unique individual aspects of depression highlight the need for a person-centred approach and continuity in care to customise treatment [46]. Furthermore, the frustration that adolescents experience in not being in control over their depression appeared to be connected to the unpredictable nature of the symptoms, varying from day to day. Other research shows that predictability plays an integral role in relation to mental health in adolescence [22]. For adolescents in general, uncertainty in social interactions is described as a barrier to experiencing mental health and feeling in control depends on external but also internal factors, which was also evident in our findings. Beyond recognising strategies and improvements in managing symptoms, validation of adolescents´ efforts and challenges might promote healthy behaviours.

The fourth aspect of health concerned experiences of attaining an inner drive, as a sense of purpose or motivation. The presence of an inner drive and the ability to find joy in the everyday embodies a hedonic perspective encompassing happiness, contentment, and an interest in life, which is denoted as ‘emotional well-being’ [48]. Adolescents from the general population have described meaningfulness as an aspect of health, while ill-health is associated with anhedonia [23]. Since anhedonia is a core symptom of depression, the desire for meaningfulness and inner drive to achieve health becomes understandable to the adolescents in our study. The adolescents also described the importance of self-compassion and self-care. Therefore, elements of motivational work and self-compassion may be beneficial in promoting health and treating depression among these adolescents.

The various aspects of health described by adolescents with depression underscore the multifaceted nature of health and suggest that a holistic approach, considering lifestyle habits, social connection, symptom management, and inner drive, is crucial for fostering their well-being and promoting health.

### Strengths & limitations

In qualitative research, trustworthiness is defined according to the four criteria of credibility, dependability, confirmability, and transferability [49].

Credibility refers to confidence in the truth of the data and the analysis. In this qualitative study, the credibility was strengthened through purposive sampling. The aim of a phenomenographic approach [30] is to identify variations. In previous phenomenographic studies, data from 20 participants have been sufficient to present varying conceptions of a specific phenomenon [31]. The study reached saturation with the sample size of 33 participants. The relatively large sample size enabled more variations and rich descriptions of adolescents’ understanding of health. The adolescents were encouraged to talk freely about health and were reminded that no answers were considered incorrect, which adds credibility [50]. To strengthen the study’s confirmability and credibility, recurrent discussions were held within the research group throughout the research process. Each researcher reflected on their background and experiences, recognizing how these might influence interpretations of adolescents’ understandings of health. An openness to the adolescents’ diverse perspectives was maintained, using open-ended questions like, ’Please, tell me more’ and ’Please give some examples’ to allow their narratives to unfold naturally. Additionally, ongoing discussions about alternative perspectives and interpretations of the empirical data were conducted, critically examining how individual experiences might shape understanding. This collaborative approach enriched the analysis and highlighted the complexity of adolescents’ conceptions of health, ensuring a more nuanced interpretation of the findings. Finally, the study’s dependability was strengthened through a comprehensive description of the research design and through gathering data [50]. Interviews were conducted via Microsoft Teams or Zoom to create a comfortable and secure environment for the adolescents, allowing them to participate from home. Video conferencing platforms offer several advantages for qualitative interviews, including convenience, ease of use, and the ability to foster personal connections [51]. Furthermore, Archibald et al. emphasize that platforms like Zoom can be just as effective as in-person interviews for qualitative data collection, thanks to their interactivity and features such as screen sharing, recording, and overall flexibility. Thus, videoconferencing serves as a valuable alternative that enhances accessibility while supporting effective qualitative research.

A limitation is that adolescents who already exercised regularly were excluded from the study. This may affect the results in different ways. Either these adolescents find exercising more important for their health, or they might believe that exercise does not affect their well-being that much since they are still suffering from depression. Since participants were interviewed after being randomised and completing exercise or leisure activities, the type of intervention for the individual might affect the view of what was considered as important for health. If someone felt less depressed after the exercise intervention, they might find physical activity more important than those who did not experience the effect of the intervention.

On the contrary, participants who improved in mood from the leisure group might consider sociability more important for their health. The adolescents’ view on health may also be affected by what is learned in school and from meeting staff during treatment for depression, for example, the brief psychosocial intervention. One adolescent wanted a parent to be present during the qualitative interview, and this might have affected the answers, but it probably led to a more comfortable setting. Even so, a greater representation of females may have influenced the outcome and, consequently, the study’s transferability; [50] however, depression is more common among females after puberty [4]. Nine male participants can, therefore, be considered a sufficient distribution of the gender demographics. Hence, this gender ratio is expected and beneficial for the study’s transferability [50].

## Conclusion & future directions

Adolescents with depression emphasized the significance of establishing sound routines by managing everyday life, together with connecting to others by having access to social resources, managing depression symptoms by having control over their mental illness, and attaining inner drive by experiencing joy in everyday life as essential aspects of health. Customising evidence-based treatments in line with personal preferences regarding these health-promoting aspects could enhance the effectiveness of treatments. As adolescents with depression have varying perspectives on health, health promotion needs to account for individual preferences. There is a need for a balanced approach, where adolescents’ perspectives are valued while evidence-based knowledge is simultaneously adopted. Future research could explore individualised health promotion as an add-on to evidence-based treatments for depression among adolescents, for example, with a focus on routines, social connections, or finding an inner drive.

## Supporting information

S1 Checklist The consolidated criteria for reporting qualitative research (COREQ 32)—item checklist. (DOCX)
